# Collective Decision-Making in Homing Pigeons: Larger Flocks Take Longer to Decide but Do Not Make Better Decisions

**DOI:** 10.1371/journal.pone.0147497

**Published:** 2016-02-10

**Authors:** Carlos D. Santos, Sebastian Przybyzin, Martin Wikelski, Dina K. N. Dechmann

**Affiliations:** 1 Department of Migration and Immuno-ecology, Max Planck Institute for Ornithology, Radolfzell, Germany; 2 Departamento de Biologia, Centro de Ciências Biológicas e da Saúde, Universidade Federal do Maranhão, Campus do Bacanga, São Luís, MA, Brazil; 3 Department of Biology, University of Konstanz, Konstanz, Germany; Arizona State University, UNITED STATES

## Abstract

Social animals routinely are challenged to make consensus decisions about movement directions and routes. However, the underlying mechanisms facilitating such decision-making processes are still poorly known. A prominent question is how group members participate in group decisions. We addressed this question by examining how flocks of homing pigeons (*Columba livia*) decide their homing direction. We released newly formed flocks varying in size and determined the time taken to choose a homing direction (decision-making period) and the accuracy of that choice. We found that the decision-making period increases exponentially with flock size, which is consistent with a participatory decision-making process. We additionally found that there is no effect of flock size on the accuracy of the decisions made, which does not match with current theory for democratic choices of flight directions. Our combined results are better explained by a participatory choice of leaders that subsequently undertake the flock directional decisions. However, this decision-making model would only entirely fit with our results if leaders were chosen based on traits other than their navigational experience. Our study provides rare empirical evidence elucidating decision-making processes in freely moving groups of animals.

## Introduction

Social animals routinely need to make decisions in common agreement with other members of their society. A diversity of consensus decision-making models can be found in nature, all of which also occur in human societies [1, 2]. Consensus can be reached in a democratic manner, through a quorum or opinion pooling, but also when only one or a few leaders are assigned to decide [2, 3]. However, depending on the model used, the quality of decisions may vary considerably [4].

It is generally assumed that the accuracy of decisions increases with the number of individuals involved in the decision-making process [2, 5, 6]. The pool of information in a group is potentially more valuable than the experience of a single individual, and opinion pooling may be a way to improve the accuracy of decisions [6, 7]. However, it has been recently shown that groups with a larger number of social interactions tend to make less accurate decisions [4]. Leadership can obviate the complexity of interactions in animal groups and lead to accurate decisions [4, 8–10], but this crucially depends on the quality of the information possessed by the leaders [4]. Leadership can also result in faster decisions, which can be highly advantageous in many circumstances, such as escaping from a predator [11], finding shelter in harsh conditions [12], or activating an emergency plan [13].

Homing pigeons (*Columba livia*) have been frequently used as a model in collective decision-making studies [14–18]. Emerging evidence indicates that leadership structures pigeon flock navigation, with most flock members following the decisions made by one or a few leaders [17–20]. Furthermore, in flocks composed of the same individuals the leadership hierarchy tends to be repeated over time [18, 19]. However, there is also evidence that pigeons may share navigational decisions when there is little conflict of opinion about the route to follow [14]. Whether pigeon flocks decide democratically or rely on leadership thus remains debatable and requires further empirical evidence.

Homing pigeons are known to spend a considerable amount of time circling at the release site before undertaking their homing trip. This behaviour has been suggested to have orientation but also social purposes [21–23]. While the social mechanisms of decision-making have been fairly well studied for the period after pigeons have left the release site [14, 16–20], the circling phase of homing flights has never been studied in detail. However, the circling phase is likely to include the most crucial decisions of a homing flight, including how the flock must be structured and which homing direction should be followed.

Our study investigates flock decision-making during the circling phase of homing flights. We built flocks of different sizes, composed of birds arbitrarily selected from a larger pool of individuals, which were then released from unfamiliar sites. This procedure prevented flocks from using previous flight experience to make decisions. We examined how the time taken to make a decision and the decision accuracy co-vary with flock size.

Similar parameters were previously used to compare flight performance between single birds and small flocks [24–26]. Keeton [25] and Benvenuti et al. [24] have shown that small pigeon flocks exhibit longer vanishing times than singletons. These two studies also showed that small flocks and singletons exhibit similar directional accuracy when leaving the release sites, but another study [26] showed that flocks take less scattered directions than single birds.

Based on these findings we hypothesized that flocks of increasing size take longer time to make flight decisions. We also hypothesized that larger flocks exhibit higher directional accuracy, because larger flocks gather a larger pool of information and thus have the potential to make better decisions. We discuss the patterns observed in the light of current theories of flock decision-making and structuring.

## Materials and Methods

### Ethics Statement

All experimental procedures were approved by the Ethical Committee of Baden-Württemberg (Regierungspräsidium Freiburg Abteilung 3) through the license number 35–9185.81/G-13/19.

### Homing experiments

Experiments were conducted with a group of 66 homing pigeons housed in one loft located in Eigeltingen (47°53'37"N, 8°55'35"E), Southern Germany. Prior to the experiments, the group was trained with other pigeons of the same loft, every week (year round), in releases from distances of up to 50 km and different homing directions. This was part of the breeder’s regular training protocol. Individuals in the experimental group varied in age and flight experience (younger birds had participated in fewer training flights then older birds). All birds that were included in the group were older than one year. We chose ten sites at a distance of 5 to 20 km around the loft for our experimental releases. None of these sites had been used in the pre-experimental training releases. We selected the sites based on optimal visibility in all cardinal directions, allowing us to observe pigeon vanishing bearings. On every release day, we arbitrarily selected 37 of the 66 pigeons in the loft and transported them to the release site in a carrying basket by car. These pigeons were then placed into groups of 2, 5, 10 and 20 pigeons in a release basket at the release site. The removal of pigeons from the carrying basket was arbitrary, with no selection for pigeon ID. The different group sizes were built and released in a randomized order at each release site. Birds remaining in the carrying basket had no visual contact with the release basket or the flying groups. For each release, we placed the release basket under the open sky and opened a wide side door after 15 minutes of habituation. We observed released flocks with 10 x 52 binoculars until they vanished from sight. We started handling the next group 15 minutes after the previous group had vanished from sight. We recorded the time from when the birds left the release basket until they stopped circling and assumed a straight course (i.e. decision-making period) and the vanishing bearing. Flocks sometimes split briefly during the decision-making period, but they always left the release area as a whole. Data analysis was conducted with the software package R, version 3.1.0 [27], and circular statistics were carried out with the package Circular [28].

### Data accessibility

All data are available at http://dx.doi.org/10.5061/dryad.k15v4.

## Results

**Fig 1:**
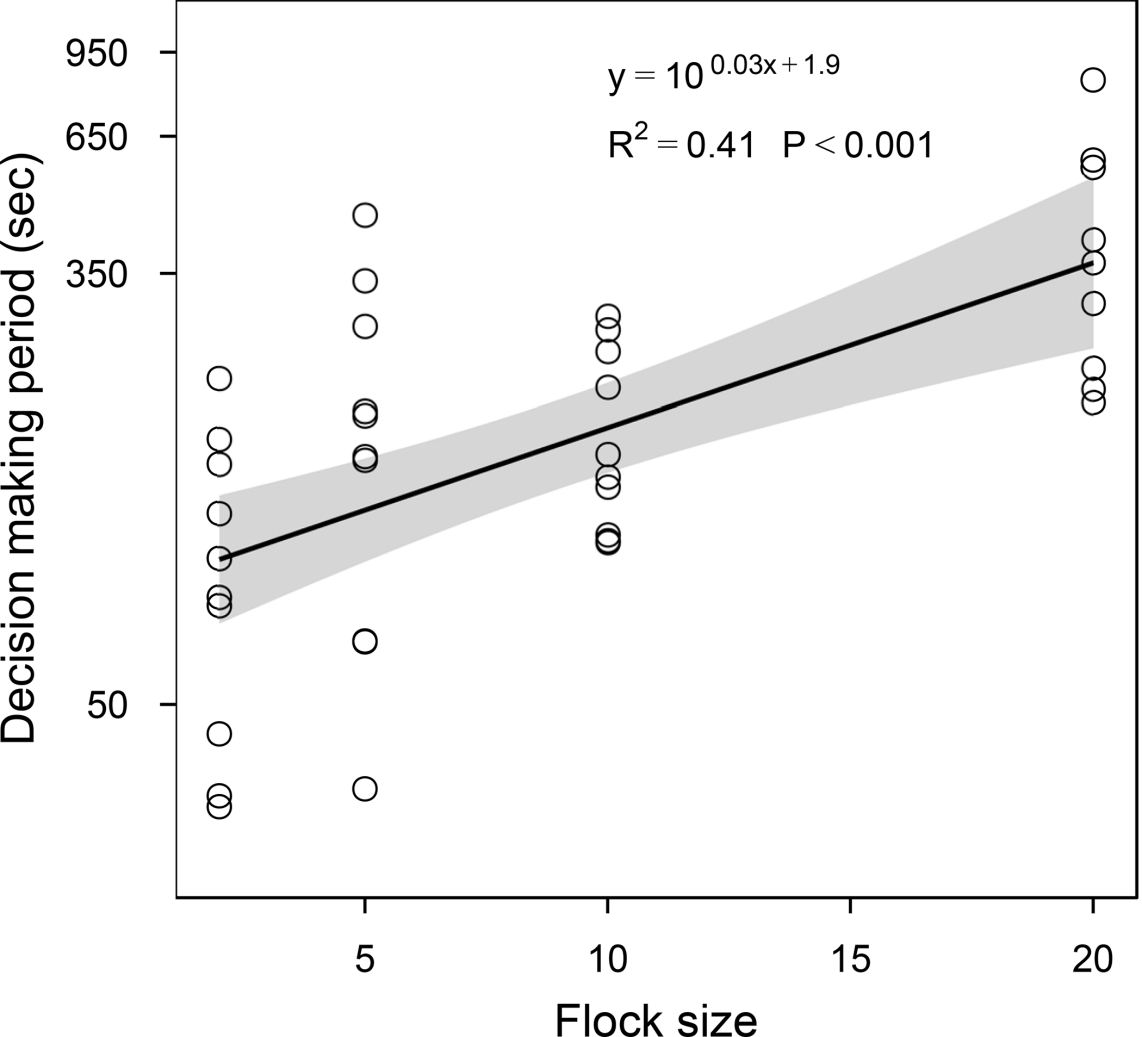
Relationship between pigeon flock size and the decision-making period (i.e. time taken to assume a straight homing direction). Note that *y* values are represented in a logarithmic scale. Shading represents regression 95% confidence intervals. Points represent individual observations.

We conducted 10 releases with each pigeon group size (2, 5, 10 and 20 individuals). One release of 20 individuals was excluded from the analysis because pigeons were disturbed by a raptor. Groups varied in the time until they assumed a straight course and left the release area (decision-making period). The decision-making period increased exponentially with the group size (Fig 1). This increase was equivalent to that expected in the number of pairwise interactions among group members. In fact, the predicted values of the relationship shown in Fig 1 were highly correlated with the number of possible linear combinations between members of the group (Pearson's correlation: R = 0.99, P < 0.001).

**Fig 2:**
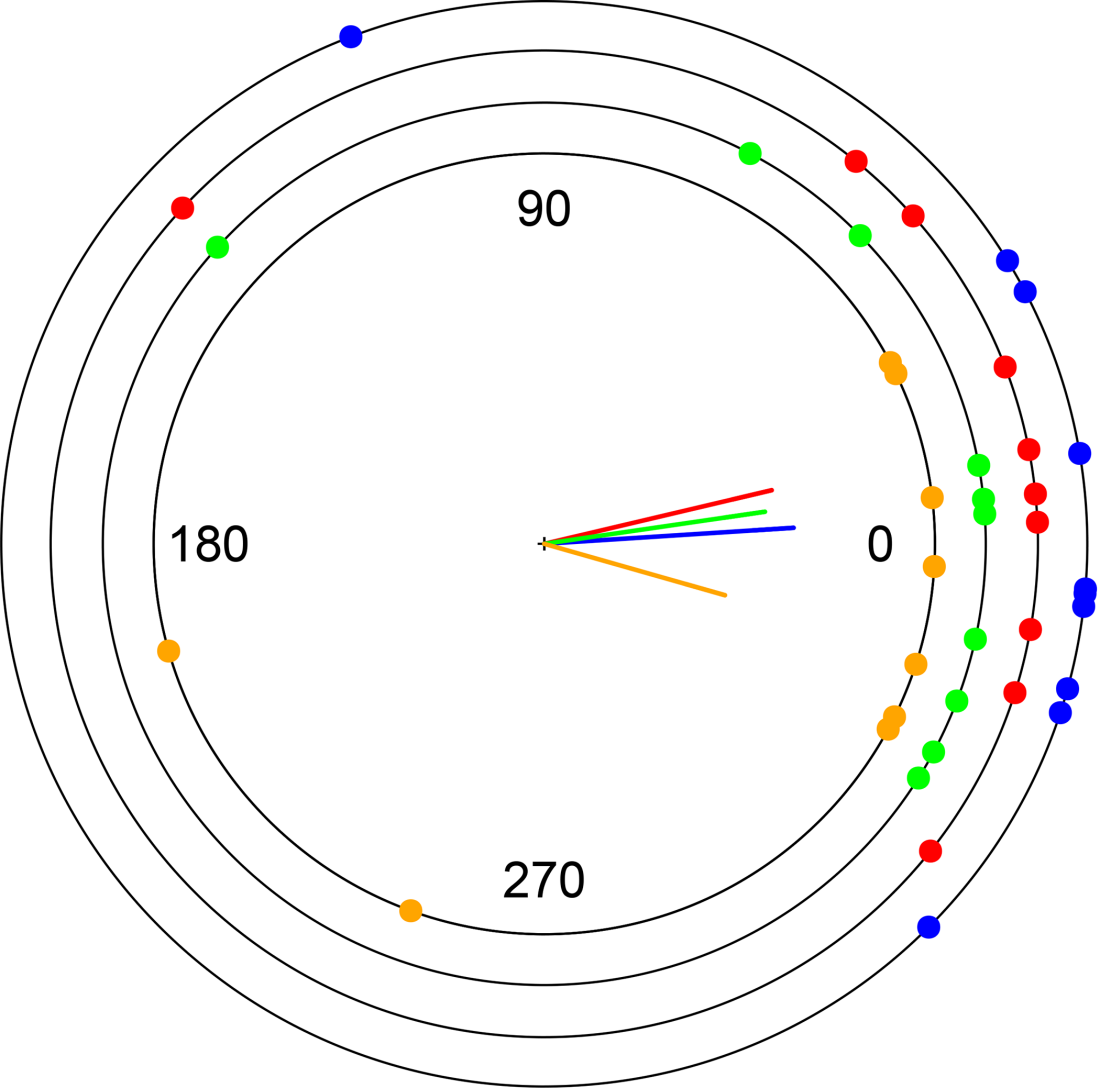
Homing directions assumed by pigeon flocks of different sizes. Correct homing direction was set to 0°. Points represent individual observations. Inner lines represent mean vector lengths for each flock size (0.80, 0.75, 0.71 and 0.60 for group sizes of 2, 5, 10 and 20 pigeons respectively). Colour codes are blue, red, green and orange for group sizes of 2, 5, 10 and 20 pigeons respectively.

All group sizes showed non-random directions at vanishing (Rayleigh test, groups of 2: P < 0.001, groups of 5: P = 0.002, groups of 10: P = 0.004 and groups of 20: P = 0.03; Fig 2), and were significantly oriented towards home (V test, P < 0.001 for groups of 2, 5 and 10 individuals and P = 0.006 for groups 20 individuals; Fig 2). The directional error was not significantly affected by the group size (Kruskal-Wallis test applied to the ranks of angular distances, following [29], χ^2^ = 0.601, P = 0.8962; Fig 2).

## Discussion

We show that the time required by pigeon flocks to choose a homing direction increases with the size of the flock. These findings are in accordance with earlier studies showing an increase in the vanishing time (time to leave the release location) from single pigeons to flocks [24, 25].

Importantly, we show for the first time that the relationship between decision-making time and flock size is exponential, which suggests the occurrence of multiple interactions between flock members during the decision-making period. In fact, the predicted values of the flock size vs decision-making time relationship were highly correlated with the number of unique paired combinations of the flock members. This evidence is consistent with the idea of a participative decision-making process occurring during the circling phase of homing flights.

Two possible participative decision-making scenarios fit with the observed patterns: (1) flocks democratically decide their homing direction, or (2) flock members democratically decide which leaders they should follow. In (1), interactions need to be established between all flock members, which would imply decision-making time to exponentially increase with flock size. In (2), the number of leader candidates is expected to increase with flock size, as well as for the number of opinion conflicts among them. Thus the time taken by flock members to elect leaders should also increase exponentially with flock size. It should be stressed that we consider the process of flock structuring (for instance the establishment of a leadership hierarchy) as part of the decision-making task.

These results also allow us to exclude the possibility of a pre-determination of leaders in the home loft. In fact, if leadership rules were clearly defined in the home loft, leaders should assume command of homing flights without being questioned. Furthermore, we may expect leaders from large flocks to orient faster than those from small flocks, because the pool of potential good leaders is larger in large flocks. Therefore, if leaders are pre-determined we may expect flock size to either have no influence on decision-making time or to promote a decrease of this parameter, but not the dramatic increase that we observed. It should be emphasized that our concept of leader pre-determination implies that all the possible conflicts are resolved in the loft and not during the homing flight.

We additionally show that the accuracy of homing directions was not affected by the size of the flock. This was also found in earlier studies [24, 25], but it contradicts the many-wrongs principle [6, 30] and the findings of Tamm [26].

According to the many-wrongs principle, information pooling from many inaccurate compasses yields a single more accurate compass because individual orientation error is suppressed by group cohesion [6, 30]. It is therefore expected that larger flocks navigate more accurately than smaller flocks [6, 30]. This principle applies to any democratic choices of moving directions carried by animal groups [6]. In this context, the absence of a relationship between flock size and directional accuracy indicates that our flocks do not decide their homing direction democratically. Therefore, the scenario (1), described above, should be rejected.

We have also suggested that the democratic process occurring during the circling phase of homing flights could be a democratic choice of flock leaders that later determine flock homing direction (scenario (2)). But would this scenario agree with the absence of a relationship between flock size and the accuracy of homing direction?

Two more specific scenarios should be considered in order to answer this question: (2.1) the leaders could be elected based on their navigation experience, or (2.2) the leaders could be elected regardless of their navigation experience. In (2.1) we should expect larger flocks to navigate more accurately because they have a larger pool of leader candidates and can potentially choose better leaders. Therefore this scenario also disagrees with the evidence from our results. In contrast, in scenario (2.2) we should not expect an effect of flock size on the accuracy of homing directions. Therefore this scenario is fully consistent with our results.

Taken together, the observed effects of flock size on the decision-making time and the absence of an effect on their directional accuracy are consistent with the idea of flocks democratically choosing leaders based on traits other than their navigational experience.

The direct observation of flock behaviour also suggests that there is a leader election during the flights. Once the flocks gained altitude and started circling, flock cohesion was frequently perturbed by the movement of individual birds in directions that slightly differed from the main flock. These birds often had a contagious effect on other flock members, and if they managed to recruit a significant proportion of the flock, the remainder would tend follow their direction. But, if only a few birds were recruited, the flock would temporarily split. This behaviour was previously described in fish shoals showing quorum decision-making [31, 32].

We should emphasize, however, that our conclusions may be specific to newly formed flocks of pigeons released from unfamiliar sites. Therefore, we cannot exclude that repeated flights with the same group retain decisions made earlier, for instance resuming leaders previously chosen. Also, flocks released from familiar sites may exhibit different patterns of navigational accuracy than that observed here. If flocks choose leaders and leader candidates are better oriented and less conflicting, it is likely that the navigation experience of leaders plays a key role for leader choice [16–18].

Pigeon flock decision-making is overall a remarkable phenomenon, illustrating the complexity of collective animal movement. The intricate collection of factors and circumstances generating this phenomenon clearly present major challenges as well as unique opportunities for future research.
